# Attentional modulation of sensory gating during a visuomotor task

**DOI:** 10.1016/j.jphyss.2026.100080

**Published:** 2026-05-15

**Authors:** Cuong Nguyen Van, Duc Le Trung, Takeru Okamoto, Masataka Tatsumi, Thu Nguyen Dang, Yuya Hyodo, Satoshi Kase, Tomoyuki Kurose, Takeshi Imura, Hisao Nishijo, Susumu Urakawa

**Affiliations:** a Department of Neurorehabilitation and Emotional Science, Graduate School of Biomedical and Health Sciences, Hiroshima University, 1-2-3 Kasumi, Minami-ku, Hiroshima City, Hiroshima 734-8553, Japan; b Graduate School of Innovation and Practice for Smart Society, Hiroshima University, 1-5-1 Kagamiyama, Higashi-Hiroshima, Hiroshima 739-8529, Japan; c Department of Neurology, Military Hospital 103, Vietnam Military Medical University, 261 Phung Hung Street, Ha Dong District, Hanoi 12108, Viet Nam; d Department of Anesthesiology, Military Hospital 103, Vietnam Military Medical University, No. 261 Phung Hung Street, Ha Dong District, Hanoi 12108, Viet Nam; e Department of Anatomy and Histology, Graduate School of Biomedical and Health Sciences, Hiroshima University, 1-2-3 Kasumi, Minami-ku, Hiroshima City, Hiroshima 734-8553, Japan; f Faculty of Human Sciences, University of East Asia, 2-12-1 Ichinomiya Gakuen-cho, Shimonoseki City, Yamaguchi 751-8503, Japan

**Keywords:** attention, sensory gating, heart rate variability, eye blink rate, motor learning

## Abstract

Attention is crucial for motor performance because it enhances task-relevant processing while suppressing distractions. However, physiological markers of attentional engagement during motor activity remain incompletely understood. The present study investigated how attention modulates sensory gating (SG) along with heart rate variability (HRV) and eye blink rate (EBR) during a visual cue-based typing task. Twenty-five participants performed under three conditions: (1) no typing, (2) free typing, (3) precision typing. The results revealed that higher attention led to a consistent increase in SG across distinct modalities. Particularly, this modulation was observed in higher-order components: N1, P2 in auditory evoked potential; P1 in visual evoked potential; P2, P3 in somatosensory evoked potential. Furthermore, task-related HRV modulation was associated with varying attentional and motor demands, while EBR decreased with increased visual attentional engagement. These findings further clarify SG and autonomic changes during attentional modulation in motor tasks, highlighting their potential for assessing attentional engagement.

## Highlights

• Multisensory gating (auditory, visual, somatosensory) was consistently modulated by attentional engagement at cortical level.
• Sensory gating modulation was observed in higher-order components: N1, P2 in AEP; P1 in VEP; P2, P3 in SEP.
• Task-related HRV was associated with varying attentional and motor demands, while EBR decreased with increased visual attentional engagement in motor task.

## Introduction

Attention is a higher-order cognitive function enabling selective focus and the suppression of irrelevant information. While it has been extensively studied in cognitive contexts, it is also essential for motor learning (ML), refinement, and efficiency [1]. Despite its importance, attentional engagement during motor activities is typically assessed using task performance measures and self-report questionnaires, which aim to estimate the level of attentional focus [2]. However, these approaches are limited by their subjectivity and their inability to capture real-time fluctuations in attentional states. In contrast, objective physiological markers —such as neurophysiological and autonomic indices (e.g., EEG-derived measures, heart rate variability, and eye-blink rate)—offer the potential to continuously and quantitatively monitor attentional engagement during ongoing behavior [3]. Nevertheless, how these physiological indices reflect attentional modulation during complex motor performance remains insufficiently understood.

Attentional states have been widely investigated using neuroimaging techniques, such as functional magnetic resonance imaging (fMRI) and magnetoencephalography (MEG) [4]. However, these approaches are often limited by constraints related to ecological validity, experimental flexibility, and/or temporal resolution [5], [6]. In contrast, electroencephalography (EEG) provides a favorable balance of high temporal resolution, cost-effectiveness, and experimental feasibility, making it well suited for capturing rapid fluctuations in attentional states [7]. Among EEG-based approaches, spectral power analysis and sensory gating (SG) are commonly used for attentional studies. SG reflects the neural suppression of irrelevant sensory inputs and serves as an index of selective attentional filtering [8]. It can be quantified using event-related potentials (ERP) elicited by distractors, where reduced amplitudes indicate more effective inhibitory processing and more efficient allocation of attentional resources [9]. Importantly, SG can be applied across various task contexts and is relatively independent of overt behavioral performance. Previous studies have demonstrated SG modulation during attentional engagement across both cognitive paradigms (e.g., oddball task, Go/No-Go task) and simple motor tasks (e.g., button-press responses) [10]. However, these paradigms typically involve single-modality distractors (auditory [11], visual [12], or somatosensory [13]). Therefore, it remains unclear how SG operates during continuous motor performance requiring sustained and complex attentional control under multimodal sensory interference. Moreover, evidence regarding the temporal dynamics of SG is inconsistent. Some studies report modulation in both early and late ERP components in complex tasks [14], [15], whereas others have observed effects mainly in later components, such as auditory P2 (∼200 ms) [16], visual P1 (∼200 ms) [17] and somatosensory P3 (∼300 ms) [18]. These discrepancies suggest that SG dynamics may depend on task complexity and attentional demand, warranting further investigation in more realistic motor contexts.

Beyond central neural measures, attention is also reflected in peripheral physiological signals, including heart rate variability (HRV), which reflects functional coupling between prefrontal cortical and subcortical systems involved in attention regulation [19], [20]. Resting HRV is linked to individual differences in attentional control [21], whereas task-related HRV typically decreases under cognitive demand, reflecting adaptive resource allocation [22], [23]. Similarly, eye blink rate (EBR) is another behavioral marker of attentional engagement linked to dopaminergic mechanisms of cognitive control [24], and reduced EBR has been observed during sustained visual tasks such as reading, film viewing, and video-based learning [25], [26], [27], likely supporting continuous visual processing by minimizing interruption from distractors [28]. However, changes in task-related HRV and EBR respond to attentional demand during complex motor tasks remain poorly understood.

Accordingly, the present study investigated how SG across modalities, together with task-related HRV and EBR, is modulated by attentional demand during complex motor performance. To this end, we designed a visually guided typing task with three attentional conditions, incorporating concurrent distractors from auditory, visual, and somatosensory modalities. We aimed to examine attentional modulation of SG across modalities and to explore changes in task-related HRV and EBR in relation to the ML process. We hypothesized that increased attentional demands would enhance SG, with concurrent reductions in task-related HRV and EBR.

## Methods

### Participants

Twenty-eight healthy volunteers (14 males, 14 females; mean age = 21.96 ± 0.92 years) from the Hiroshima University community were initially recruited. The required sample size was calculated with G*Power version 3.1 [29] for a within-subject design with three conditions, aiming primarily to detect the main effects of the condition. On the basis of a previously reported effect size of f = 0.3 [30], an alpha level of 0.05, and a desired power of at least 0.8, the calculated required sample size was ≥ 20. All individuals were strongly right-handed, as confirmed by the Edinburgh Handedness Questionnaire.

The inclusion criterion for participants was no history of neurological, psychiatric, or musculoskeletal disorders. The participants were instructed to abstain from caffeine or analgesics for 24 h prior to testing and were excluded if they reported fatigue or insomnia. Three participants were excluded because of excessive EEG signal noise caused by technical issues, resulting in a final sample of 25 participants (13 males, 12 females; mean age = 21.88 ± 0.92 years).

The study was approved by the Ethics Committee of Hiroshima University (E2024–0237) and conducted in accordance with the Declaration of Helsinki and the U.S. Code of Federal Regulations for the protection of human research participants. All participants provided written informed consent prior to the study.

### Task design

**Fig. 1:**
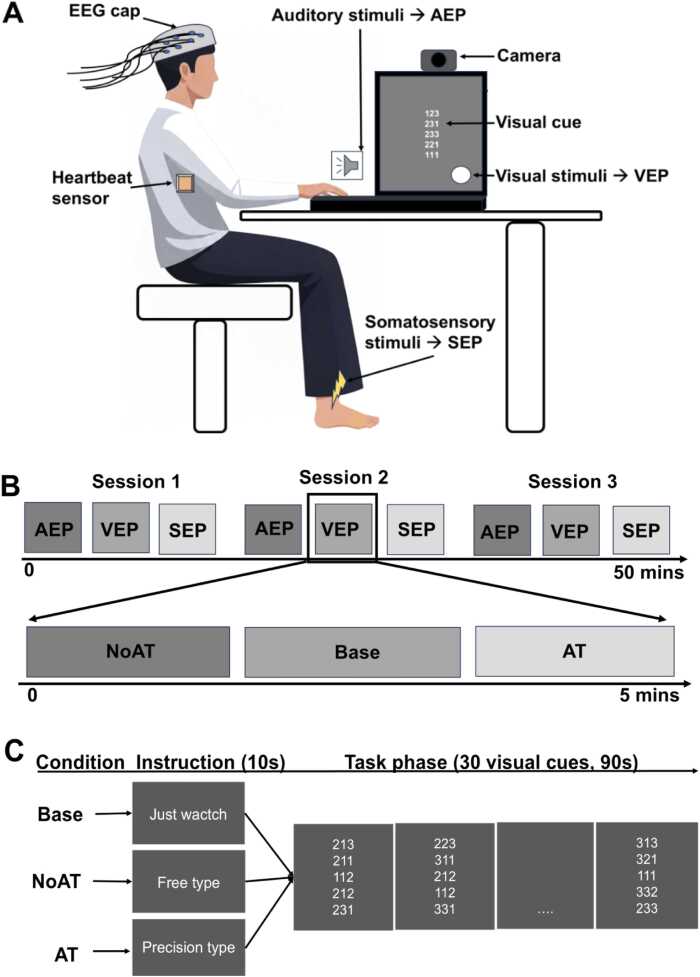
**(A)** The participant sat on a chair, wearing an EEG cap and heartbeat sensor, performing a visual cue typing task on a monitor while receiving one type of irrelevant stimulus: auditory (via speaker), visual (at peripheral site of the monitor where visual cues displayed), or somatosensory (transcutaneous electrical stimulation of the tibial nerve at the ankle). (**B)** The task consisted of three sessions, each containing three blocks corresponding to the three irrelevant stimulus types. Each block included three trials representing three different attentional conditions including Base, NoAT, and AT. **(C)** Each trial comprised 10 s of instructions on the screen, including “Just watch,” “Free type,” and “Precision type.” followed by a 90-second task phase, including 30 visual cues. These instructions and visual cues were displayed on the monitor. AEP: auditory evoked potential, VEP: visual evoked potential, SEP: somatosensory evoked potential. Base: baseline, NoAT: no attention, AT: attention.

The participants performed a typing task in response to visual cues and on-screen instructions, using their right index, middle, and ring fingers to press the keys on the keyboard corresponding to the digits “1”, “2”, and “3” of visual cue, respectively (Fig. 1A).

**Fig. 2:**
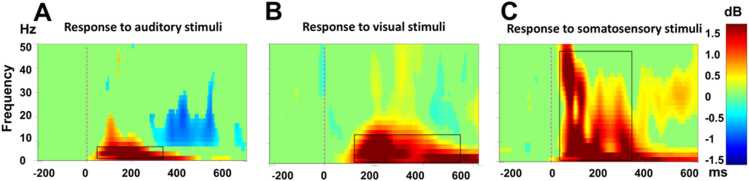
Event related spectral perturbations analysis in the Base condition showed distinct patterns for each irrelevant stimuli: auditory stimuli induced strong activity at 3–7 Hz, from 50 to 350 ms after the onset of stimuli (rectangle line) **(A),** visual stimuli induced strong activity at 3–12 Hz, from 100 to 600 ms after the onset of stimuli (rectangle line) **(B)**, and somatosensory stimuli induced strong activity at 0.5–45 Hz, from 60 to 400 ms after the onset of stimuli (rectangle line) **(C)**.

We designed the typing task in three attentional conditions: the baseline condition (Base), where participants were exposed only to visual cues without engagement; the no attention condition (NoAT), in which typing was performed without visual attention or explicit accuracy requirements, reflecting minimal additional attentional demands beyond motor execution; and the attention condition (AT), which emphasized typing accuracy, require high coordination between visual attention and motor execution. Increasing accuracy constraints may elevate both attention and difficulty; therefore, the physiological changes observed in the precision-demanding condition are interpreted primarily as reflecting enhanced attentional engagement, while acknowledging potential overlap with general task demand.

### Experimental setup and procedure

The participants were seated with their eyes 50–60 cm away from a laptop monitor (HP V194, Hewlett-Packard, USA; 18.5", 1366 × 768 pixels, 60 Hz) wearing an EEG cap and a chest heartbeat sensor (myBeat; Union Tool Co., Tokyo, Japan). To minimize artifacts and movement interference, participants kept their left hand resting on the table and were instructed to fixate on the screen.

During the task phase, the participants were passively exposed to irrelevant stimuli. Auditory and visual stimuli were generated via PsychoPy3 software (V2025.1.1, University of Nottingham, UK), whereas somatosensory stimuli were generated via an electronic stimulator (SEN-7203, Nihon Kohden, Japan). To evoke AEP, a 500 Hz, 100 ms tone was presented via a loudspeaker at 50 cm at 40 dB sound pressure level measured at ear level [14]. To evoke VEP, a white circle (2° visual angle) was presented at 15° from the center of the visual field for 230 ms [31] at one of four locations (upper/lower left or right). To evoke SEP, transcutaneous electrical stimulation (0.5 ms square pulses) was applied to the left tibial nerve at the ankle. The intensity was individually adjusted to produce a clear but tolerable sensation without muscle twitches, following a previous study [15]. Adhesive electrodes were placed 3 cm above and below the ankle. Stimulation began at 1 mA, increased until toe twitching, and then slightly decreased (mean: 6.58 ± 0.6 mA). The interstimulus intervals were 2–4 s.

Each participant performed 3 sessions, each consisting of 3 blocks of irrelevant stimuli (AEP, VEP, and SEP). A 90-second rest time was provided between sessions. Within each block, one irrelevant stimulus was presented, with 3 trials corresponding to 3 conditions: Base, NoAT, and AT. Therefore, each participant completed 27 trials, including 9 Base trials, 9 NoAT trials, and 9 AT trials. The participants passively viewed visual cues to establish a baseline for ERP recording and a resting state for measuring HRV and EBR. NoAT involves typing without accuracy constraints, assessing physiological changes during motor execution with minimal attention demands. In contrast, AT required accurate typing according to the presented numbers, eliciting increased attentional engagement. These conditions were presented in random order (Fig. 1B).

Each trial began with an instruction “Just watch” in Base, “Free type” in NoAT, and “Precision type” in AT. This was followed by 30 visual cues presented over 90 s (Fig. 1 C). Each cue comprised five horizontal rows of three randomized digits (1, 2, and 3), totaling 15 digits per cue**,** subtending ∼3° × 5° of the visual angles. The digit size and spacing were ∼0.5°, and the digit was presented in white (Fig. 1 A).

### Motor performance recording and analysis

Motor performance was evaluated by typing data acquired via PsychoPy3 software under NoAT and AT. Typing speed was calculated by the total number of digits entered, regardless of accuracy. The precision typing results in AT were calculated as the percentage of correctly typed digits relative to the total number of digits presented. The linear slope of the precision results across trials was used as a motor learning index (MLI), which was calculated via the least squares method to fit a linear regression line to the data.

### SG data recording and analysis

EEG data were recorded using an ActiveTwo system (BioSemi Inc., Amsterdam, Netherlands) with six active Ag/AgCl electrodes (O1, O2, Oz, C1, C2, Cz) positioned according to the international 10–20 system. The common mode sense and driven right leg electrodes served as the reference and ground, respectively. The direct current offset was kept below ±20 μV. The signals were acquired at a sampling rate of 2048 Hz and bandpass filtered online between 0.16 and 100 Hz.

Offline processing was performed in EEGLAB version 2024.0 and ERPLAB version 10.1 toolboxes implemented in MATLAB R2019a (MathWorks, Natick, MA, USA). The raw EEG data from the Base were first segmented into groups corresponding to each stimulus and then downsampled to 256 Hz. The data were bandpass filtered from 0.5 to 50 Hz via a zero-phase, FIR filter and notch filtered at 50 Hz to remove power-line noise. Re-referencing was performed for the linked mastoids. Artifacts were removed via a combination of visual inspection and independent component analysis with the Infomax algorithm. The components were classified via the independent component label plugin, and those labeled nonbrain sources (eye, muscle, heart, line noise, channel noise, or other) with a classification probability > 0.7 were rejected (between 1 and 3 components per participant). The data were then segmented into epochs time-locked to stimulus onset, ranging from −500 to 1000 ms, with the baseline corrected from −500 to 0 ms. A total of 90 epochs for each stimulus under each condition were averaged.

Epochs were then decomposed into time–frequency representations via a complex Morlet wavelet transform with cycles starting at 1 and increasing by a 0.8 power scale factor across frequencies from 0.5 to 50 Hz, with a resolution of 2 Hz, to compute event-related spectral perturbations. On the basis of the result of that method and reference to prior ERP studies [13], [14], [32], frequency bands associated with sensory responses were identified: auditory (3–7 Hz, 50–350 ms) (Fig. 2 A), visual (3–12 Hz, 100–600 ms) (Fig. 2 B), and somatosensory (0.5–45 Hz, 60–400 ms) (Fig. 2 C). Corresponding bandpass filters were subsequently applied to all conditions for further ERP analysis.

For ERP quantification across all conditions, downsampling, bandpass filtering, and artifact removal were performed as described above prior to redefining the epochs from −200 −800 ms relative to stimulus onset with baseline correction from −200 −0 ms. Artifacts were further screened in ERPLAB via automated voltage thresholds (±75 μV), moving window peak—peak detection, step—like change detection, and blink rejection. The remaining clean epochs were averaged separately for each condition and modality (auditory, visual, and somatosensory). Peak latencies were identified from grand-average waveforms in the Base, and mean amplitudes were extracted within those latency ranges for each participant. The following ERP components were analyzed: AEP: P1 (30–70 ms), N1 (110–150 ms), P2 (210–250 ms), and N2 (310–350 ms) at Cz; VEP: N1 (90–130 ms), P1 (170–210 ms), N2 (240–280 ms), and P2 (410–450 ms) at Oz; and SEP: N1 (60–100 ms), P2 (180–220 ms), and P3 (270–310 ms) at Cz. To quantify the overall magnitude of the SG within each modality, composite amplitude indices were computed as follows [14]: total AEP = P1 + P2 − N1 − N2, total VEP = P1 + P2 − N1 − N2, total SEP = P2 + P3 − N1.

### HRV data recording and analysis

HRV was recorded at a sampling rate of 1000 Hz. The data were subsequently segmented into trial-based windows and categorized according to experimental conditions and rest periods. Offline analyses were also conducted in MATLAB R2019a using the PhysioNet Cardiovascular Signal Toolbox. The implausible R-R interval (RRI) (<300 ms or >2000 ms) and artifacts were manually corrected via cubic spline interpolation. The cleaned RRI series was resampled at 4 Hz to produce an evenly spaced signal for spectral analysis.

The power spectral density (PSD) was estimated using Welch’s method with 64-s windows, 50% overlap, and a Hamming window. The 64-s window was selected as a compromise between the frequency resolution and the short recording duration, providing sufficient segments for averaging while enabling separation of the low-frequency (LF) (0.04–0.15 Hz) and high-frequency (HF) (0.15–0.4 Hz) bands. PSD values were expressed in absolute units (ms²). The time-domain indices included the mean RRI, NN50 (the number of successive RRIs differing by more than 50 ms), and root mean square of successive differences (rMSSD). The 90-second recording window was selected to match the duration of each task block, enabling HRV assessment during continuous task performance without disrupting the experimental flow. Given the relatively short recording duration, frequency-domain indices were interpreted cautiously and were primarily used for within-subject comparisons across conditions rather than absolute physiological inference.

### EBR recording and analysis

EBR was recorded via an action camera (OSMO Action; DJI, China) positioned at the eye level, with the front camera recording at 1080p resolution and 60 frames/s. A blink was defined as a complete, rapid closure and reopening of both eyelids, including distinct closing, and opening phases. The partial or incomplete eye closures were excluded. Successive blinks occurring within a short temporal window were considered single events. Recordings were manually synchronized with task intervals to allow precise segmentation by condition, block, and trial**.**

### Statistical analysis

All analyses and graphs were performed using GraphPad Prism 8 (GraphPad Software, San Diego, CA, USA) and R.

To examine the effects of interaction condition × modality on SG across modalities, a linear mixed-effects model was conducted. Given that ERP components differ across modalities, analyses were performed on total amplitudes normalized to the Base (Base =1). Significant main effects were followed by Bonferroni-corrected paired *t*-tests.

To examine the effect of condition on SG within each modality, one-way repeated-measures analysis was employed. First, the normality of the dependent variables was assessed using the Shapiro–Wilk test. For normally distributed variables (p > 0.05), one-way repeated-measures analysis of variance (ANOVA) were conducted to examine the effect of condition. Sphericity was assessed using Mauchly’s test, and Greenhouse–Geisser correction was applied when the assumption was violated. Effect sizes for ANOVA were reported as partial eta squared (η²p). Significant main effects were followed up with Bonferroni-corrected paired *t*-tests, with Cohen’s d reported as the effect size. For variables that violated normality assumptions (p ≤ 0.05), repeated-measures analysis was performed using Friedman tests, with Kendall’s W (W) reported as the effect size. Significant main effects were followed up with the Bonferroni-corrected Wilcoxon signed-rank test, with effect sizes (r) reported. Trial-order effects in AT were analyzed using the same approach.

Correlations between the MLI and the modulation of SG, HRV, and EBR were assessed via Pearson’s test when variables were normally distributed and via Spearman’s test otherwise. The modulation was calculated as relative changes in AT from Base (e.g., (Base − AT)/Base). To increase the reliability of the correlation estimates, bootstrap resampling (2000 iterations) was applied to calculate 95% confidence intervals (CI). Bonferroni correction was applied to adjust the significance threshold for the multiple correlation tests. All reported p corrected (p_corr_) values were two-tailed.

## Results

### Motor performance

Motor output was controlled across the NoAT and AT to minimize potential confounding effects on HRV and SG comparisons. Participants were instructed to maintain an equivalent typing speed in both conditions, thereby limiting differences in motor-related demands that could influence hemodynamic responses, respiration, or electromyographic activity. A paired *t*-test confirmed no significant difference in typing speed between conditions (p = 0.498), indicating comparable motor execution across conditions.

**Fig. 3:**
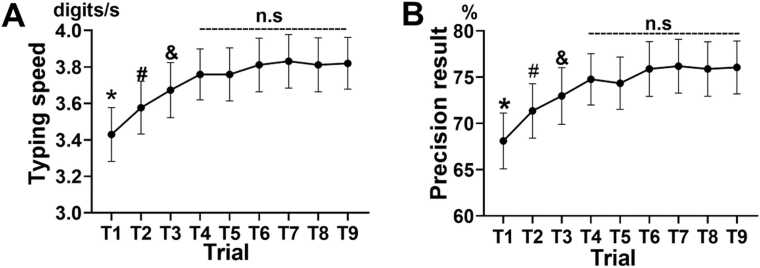
**(A)** Grand average typing speed across trials in the AT. **(B)** Grand average of precision result (calculated as the number of precise typed digits divided by total digits displayed) across trials in the AT. One-way repeated-measures ANOVA was used to examine the main effect of trial order in the AT, and post hoc paired *t*-tests with Bonferroni correction confirmed significant differences (p_corr_ < 0.05). The data are presented as means ± standard errors of the means. “*” indicates a significant difference between trial 1 and trial 4 – 9; “#” indicates a significant difference between trial 2 and trial 4 – 9; “&” indicates a significant difference between trial 3 and trial 4 – 9; and “n.s.” indicates a nonsignificant difference between trial 4 – trial 9; AT: attention condition.

In AT, one-way repeated-measures ANOVA revealed a significant main effect of trial order on both typing speed (F = 18.48, p < 0.001, η^2^_p_ = 0.435) (Fig. 3 A) and precision (F = 17.13, p < 0.001, η^2^_p_ = 0.416) (Fig. 3B). Paired *t*-tests revealed a significant increase in precision from trial 2 to trial 9 relative to trial 1 (p_corr_ < 0.001), trial 4 to trial 9 relative to trial 2 (p_corr_ < 0.001), and trial 4 to trial 9 relative to trial 3 (p_corr_ < 0.05). No significant differences were observed between trial 4–8 and trial 9 (p_corr_ > 0.05), indicating that performance plateaued after the first four trials. Accordingly, the MLI was computed as the slope of precision typing from trial 1 to trial 4.

### SG modulation

#### AEP

**Fig. 4:**
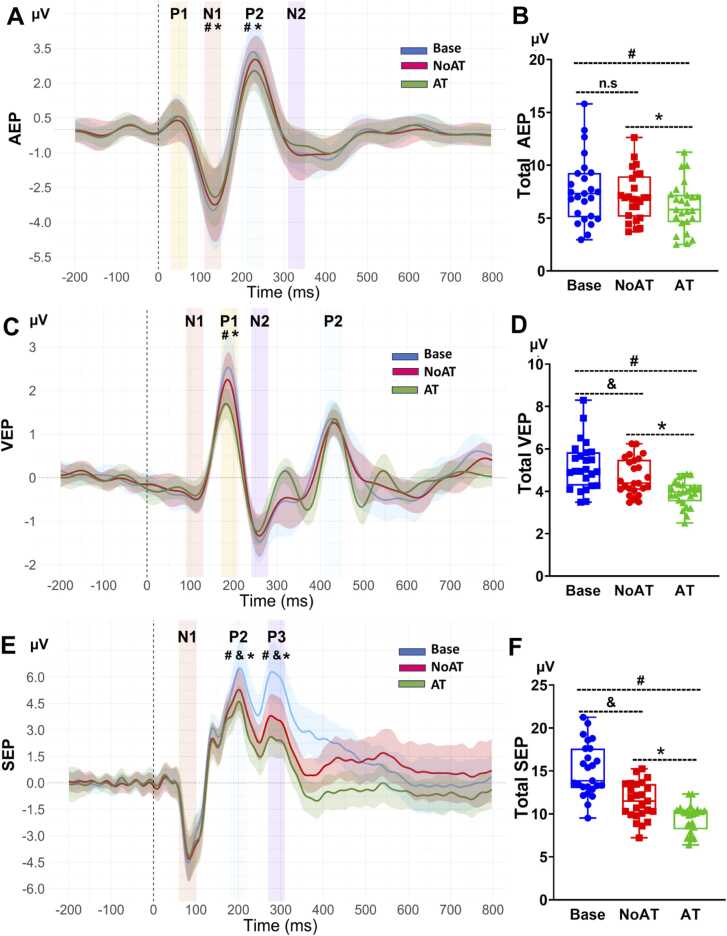
**(A)** Grand average of the AEP amplitude, P1 peak (30–70 ms), N1 peak (110–150 ms), P2 peak (210–250 ms), and N2 peak (310–350 ms). **(C)** Grand average of the VEP amplitude, N1 peak (90–130 ms), P1 peak (170–210 ms), N2 peak (240–280 ms), and P2 peak (410–450 ms). **(E)** Grand average of SEP amplitude, N1 peak (60–100 ms), P2 peak (180–220 ms), and P3 peak (270–310 ms). The data are presented as the means ± standard deviations. **(B, D, F)** Comparison of total AEP (P1 + P2 – N1 – N2), total VEP (P1 + P2 – N1 – N2), and total SEP (P2 + P3 – N1) between conditions. One-way repeated measures ANOVA was used to examine the main effect of condition, and post hoc paired t tests with Bonferroni correction confirmed significant differences (p_corr_ < 0.05). The data are presented in Box and whiskers. “#” indicates a significant difference between AT and Base condition; “*” indicates a significant difference between AT and NoAT condition; and “&” indicates a significant difference between NoAT and Base condition; AEP: auditory evoked potential; VEP: visual evoked potential; SEP: somatosensory evoked potential; SG: sensory gating; Base: baseline; NoAT: no attention, AT: attention.

The grand average of AEP amplitude was illustrated in Fig. 4A with four peaks presenting the auditory cortical response to auditory irrelevant stimuli. Generally, one-way repeated-measures ANOVA revealed a significant main effect of condition on total AEP (p < 0.001), and post hoc tests found AT elicited significant reduction compared with NoAT (p_corr_ < 0.001) and Base (p_corr_ < 0.001) (Fig. 4B, Table 1).

**Table 1:** Modulation of SG across conditions in individual peaks. One-way repeated-measures ANOVA was used to examine the main effect of condition, and post hoc paired *t*-tests with Bonferroni correction confirmed significant differences (p_corr_ < 0.05). The data are presented as the means ± standard deviation. AEP: auditory evoked potential; VEP: visual evoked potential; SEP: somatosensory evoked potential; SG: sensory gating; Base: baseline condition; NoAT: no attention condition; AT: attention condition.

Index	Condition			Main effect (F, η^2^_p_, p)	Paired *t*-test (t, Cohen’s d, p_corr_)		
	Base (1)	NoAT (2)	AT (3)		(1,2)	(1,3)	(2,3)
AEP (µV)							
P1	0.39 ± 0.90	0.26 ± 0.83	0.44 ± 0.62	1.31, 0.051 0.278	1.279, 0.25 0.639	0.366, 0.07 > 0.999	1.632, 0.32 0.347
N1	-3.19 ± 1.51	-2.97 ± 1.46	-2.65 ± 1.20	9.65, 0.287 < 0.001*	1.788, 0.53 0.259	4.000, 0.8 0.001*	2.834, 0.57 0.027*
P2	3.09 ± 1.10	2.8 ± 0.96	2.33 ± 0.81	11.23, 0.319 < 0.001*	1.698, 0.33 0.362	4.272, 0.84 < 0.001*	4.011, 0.8 0.001*
N2	-0.96 ± 0.69	-1.03 ± 1.01	-0.66 ± 0.74	2.64, 0.099 0.093	0.322, 0.06 > 0.999	2.203, 0.44 0.112	2.276, 0.45 0.096
Total	7.64 ± 3.14	7.07 ± 2.29	6.09 ± 2.99	11.66, 0.327 < 0.001*	1.633, 0.32 0.346	4.172, 0.83 0.001*	4.189, 0.84 0.001*
VEP (µV)							
N1	-0.44 ± 0.26	-0.41 ± 0.25	-0.32 ± 0.29	1.41, 0.055 0.255	0.444, 0.08 > 0.999	1.443, 0.29 0.486	1.178, 0.24 0.750
P1	2.20 ± 0.70	1.93 ± 0.58	1.39 ± 0.31	27.13, 0.531 < 0.001*	2.482, 0.49 0.061	6.496, 1.29 < 0.001*	5.382, 1.07, < 0.001*
N2	-1.30 ± 0.42	-1.19 ± 0.40	-1.06 ± 0.28	3.44, 0.12 0.049*	1.542, 0.31 0.408	2.289, 0.46 0.093	1.373, 0.28 0.547
P2	1.25 ± 0.07	1.13 ± 0.05	1.35 ± 0.06	1.12, 0.044 0.330	1.491, 0.29 0.447	1.218, 0.22 0.705	0.037, 0.07 > 0.999
Total	5.21 ± 1.15	4.68 ± 0.85	3.91 ± 0.61	24.66, 0.507 < 0.001*	3.149, 0.63 0.013*	6.077, 1.21 < 0.001*	4.442, 0.89 < 0.001*
SEP (µV)							
N1	-3.30 ± 0.95	-3.24 ± 1.12	-3.06 ± 1.35	0.59, 0.024 0.543	0.273, 0.06 > 0.999	1.245, 0.25 0.665	0.695, 0.13 > 0.999
P2	5.85 ± 1.28	4.78 ± 1.07	4.01 ± 0.89	28.87, 0.546 < 0.001*	5.392, 1.08 < 0.001*	6.283, 1.25 < 0.001*	3.364, 0.67 0.007*
P3	5.92 ± 1.50	3.5 ± 1.18	2.38 ± 0.97	93.78, 0.798 < 0.001*	11.54, 2.31 < 0.001*	11.47, 2.29 < 0.001*	4.242, 0.85 0.001*
Total	15.08 ± 3.06	11.53 ± 2.11	9.46 ± 1.59	100.7, 0.807 < 0.0001*	8.851, 1.77 < 0.001*	11.84, 2.36 < 0.001*	6.708, 1.34 < 0.001*

Regarding the individual components, the modulation of AEP amplitude across conditions did not occur in the initial component (P1), but was observed in the later components (N1 and P2). The results revealed significant main effect of condition on N1 (p < 0.001) and P2 (p < 0.001), whereas no significant effects were found for P1 (p = 0.278) or N2 (p = 0.093). The post hoc tests also found the reduction in AT compared with NoAT in N1 (p_corr_ = 0.027), P2 (p_corr_ = 0.001) and with Base in N1 (p_corr_ = 0.001), P2 (p_corr_ < 0.001) (Fig. 4A, Table 1).

#### VEP

The grand average of VEP was illustrated in Fig. 4C, with four peaks presented visual cortical response to visual irrelevant stimuli. The result revealed a significant main effect of condition on total VEP (p < 0.001) (Fig. 4D), and post hoc tests found AT elicited significant decrease compared with NoAT (p_corr_ < 0.001), and Base (p_corr_ < 0.001). Interestingly, NoAT also caused significant reduction of total VEP compared with Base (p_corr_ = 0.012) (Fig. 4C, Table 1).

More insights into individual components show the change in VEP amplitude across conditions also did not occur in the first peak N1, but was observed in later components (P1 and N2). The results revealed a significant main effect of condition on P1 (p < 0.001), and N2 (p = 0.049), whereas no significant effects were found on N1 (p = 0.255) or P2 (p = 0.33). However, the post hoc tests found that AT elicited significant reduction only in P1 compared with NoAT (p_corr_ < 0.001) and Base (p_corr_ < 0.001) (Fig. 4 C).

#### SEP

The grand average of SEP was illustrated in Fig. 4E, with three peaks presented somatosensory cortical response to electrical irrelevant stimuli. The results revealed a significant main effect of condition on total SEP (p < 0.001), and post hoc tests found significant reduction in AT compared with NoAT and Base (p_corr_ < 0.001). Interestingly, significant differences were also observed between NoAT and Base (p_corr_ < 0.001) (Fig. 4F).

Regarding the individual components, the change in SEP amplitude across conditions was not observed in the initial component (N1), but clearly seen in later components (P2, and P3). The results revealed a significant main effect of condition on P2 (p < 0.001), and P3 (p < 0.001), whereas no significant effect was found on N1 (p = 0.557). The post hoc tests found a significant decrease in AT compared with NoAT in P2 (p_corr_ = 0.007), and P3 (p_corr_ < 0.001), and with Base in P2 (p_corr_ < 0.001) and P3 (p_corr_ < 0.001). Notably, significant differences were also observed between NoAT and Base for P2 (p_corr_ < 0.001), P3 (p_corr_ < 0.001) (Fig. 4E, Table 1).

Moreover, the results revealed a significant Condition × Modality interaction (p < 0.001). However, despite these modality-specific patterns, the magnitude of the attentional effect (NoAT → AT) was comparable across modalities (AEP: 0.155, VEP: 0.141, SEP: 0.134), with all reductions reaching statistical significance (p_corr_ < 0.001) Fig. S1. These results suggest that the attentional modulation is relatively consistent across sensory modalities.

**Fig. 5:**
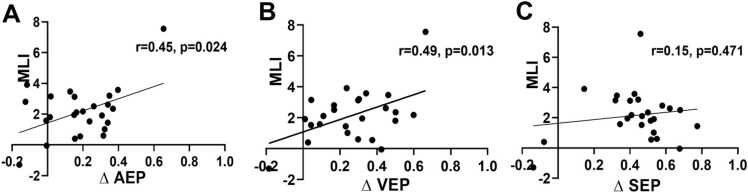
Correlations between the modulation of SG and MLI, between ΔAEP and MLI **(A)**, between ΔVEP and MLI **(B)**, between ΔSEP and MLI **(C)**. Pearson's or Spearman's correlation test was used. Nominal correlations were observed at the uncorrected level; however, none survived Bonferroni correction for multiple comparisons. Modulation of SG (ΔAEP, ΔVEP, and ΔSEP) was quantified as the relative change in the amplitudes of AEP, VEP, and SEP from the Base to the AT condition (i.e., (Base – AT)/Base). AEP: auditory evoked potential; VEP: visual evoked potential; SEP: somatosensory evoked potential; SG: sensory gating; MLI: motor learning index; Base: baseline; AT: attention.

With respect to the effect of SG modulation on ML, the results revealed that MLI showed nominal correlations with ΔAEP (Pearson, r = 0.45, p = 0.024, 95% CI [-0.288, 0.784]) (Fig. 5A), ΔVEP (Pearson, r = 0.49, p = 0.013, 95% CI [-0.062, 0.749]) (Fig. 5B), and ΔSEP (Spearman, r= 0.15, p = 0.471, 95%CI [-0.259, 0.515]) (Fig. 5C). However, none of these associations survived Bonferroni correction for multiple comparisons or bootstrap adjustment. Therefore, these findings should be considered exploratory.

### HRV and EBR modulation

Regarding resting HRV, the Wilcoxon signed-rank test revealed no significant differences between resting state and Base across all indices, including heart rate (HR) (p = 0.75), RRI (p = 0.86), NN50 (p = 0.95), rMSSD (p = 0.71), LF power (p = 0.71), HF power (p = 0.79), and total HRV power (p = 0.83). These findings suggest that, under the Base, participants remained in a near-resting physiological state, despite exposure to visual cues and distractors (Fig. S2).

Subsequently, the result revealed the modulation of HRV following the shift of attention on task, mainly in rMSSD, LF power, HF power, and total HRV power. In the time domain, the results revealed significant main effects of condition on RRIs (p = 0.04), NN50 (p = 0.004) and rMSSD (p < 0.001). However, post hoc tests revealed a significant reduction in rMSSD in AT compared with NoAT (p_corr_ = 0.003).

**Fig. 6:**
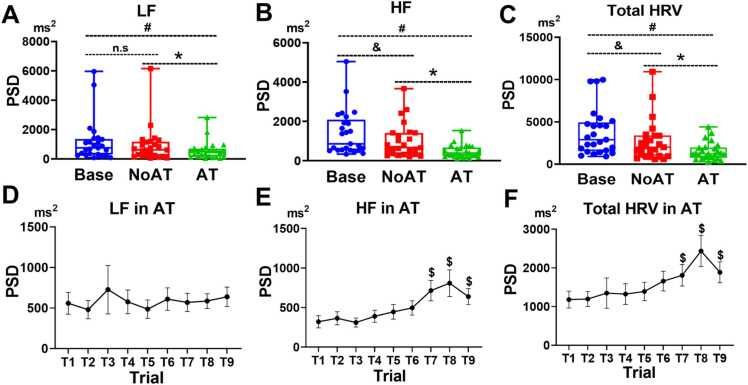
Modulation of HRV across conditions **(A–C)** and across trials in the AT condition **(D–F)**. Friedman test was used to examine the main effects of condition and trial order in AT condition, and Wilcoxon signed-rank tests with Bonferroni correction confirmed significant differences (p_corr_ < 0.05). The data are presented as means ± standard errors of means. “#” indicates a significant difference between AT and Base condition; “&” indicates a significant difference between NoAT and Base condition; “*” indicates a significant difference between AT and NoAT condition; and “$” indicates a significant difference between trial “n” and trial 1 in the AT condition; HRV: heart rate variability; LF: low-frequency; HF: high-frequency; PSD: power spectral density; Base: baseline; NoAT: no attention; AT: attention.

In contrast, frequency domain indices showed dramatic changes across conditions. The result revealed main effect of condition on LF power (p < 0.001), HF power (p < 0.001), and total HRV power (p < 0.001). Post hoc tests revealed significant reductions in AT compared with NoAT in LF power (p_corr_ < 0.001), HF power (p_corr_ = 0.002), and total HRV power (p_corr_ = 0.003). (Fig. 6 A, B, C).

The result also revealed significant modulation across trials in the AT, HRV increased in the late phase of ML progress. The results showed a significant main effect of trial order on HF power (p = 0.004) and total HRV power (p < 0.001). Post hoc tests revealed increases in HF power in trial 7 (p_corr_ = 0.001), trial 8 (p_corr_ = 0.001), and trial 9 (p_corr_ = 0.034) in comparison with first trial. Similarly, the total HRV power increased in trial 7 (p_corr_ = 0.04), trial 8 (p_corr_ = 0.001), and trial 9 (p_corr_ = 0.034) in comparison with trial 1 (Fig. 6 D, E, F).

**Fig. 7:**
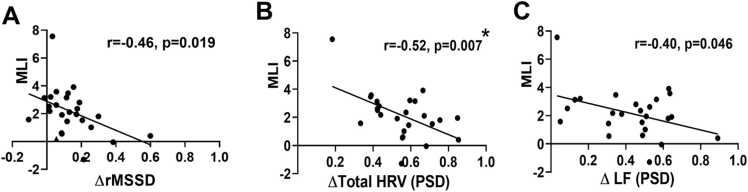
Correlations between the modulation of the HRV and MLI, between ΔrMSSD and MLI **(A)**, between Δtotal HRV power and MLI (**B**), between ΔLF power and MLI **(C)**. Pearson’s or Spearman’s correlation tests were applied as appropriate. Nominal significance was defined at p < 0.05 (uncorrected). Only the association between Δtotal HRV and MLI remained statistically significant after Bonferroni correction and bootstrap adjustment. Modulation of HRV (ΔrMSSD, Δtotal HRV, and ΔLF) was quantified as the relative change from the Base to the AT condition (i.e., (Base – AT)/Base). HRV: heart rate variability; MLI: motor learning index; LF: low-frequency; HF: high-frequency; PSD: power spectral density.

With respect to HRV modulation and ML ability, MLI showed nominal negative correlations with ΔrMSSD (r = –0.46, p = 0.019) and ΔLF power (r=-0.40, p=0.046), whereas no significant association was observed for ΔHF power (p = 0.359). A stronger negative association was found for Δtotal HRV power (r = –0.52, p = 0.007), which remained statistically significant after Bonferroni correction and bootstrap adjustment (Fig. 7). Thus, only the relationship between Δtotal HRV power and MLI can be considered statistically robust.

Similar to HRV, EBR was compared between the resting and the Base, and no significant differences were observed (p = 0.33). This suggests that the visual cues in the Base did not induce meaningful attentional engagement, indicating that Base may serve as a comparable baseline.

**Fig. 8:**
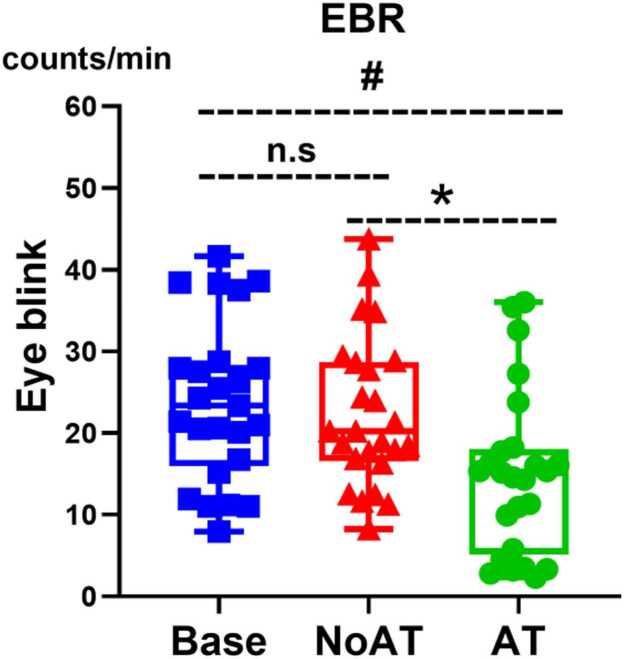
EBR modulation across conditions. Friedman test was used to examine the main effects of condition, and Wilcoxon signed-rank tests with Bonferroni correction confirmed significant differences (p_corr_ < 0.05). # indicates significant difference between AT and Base conditions. n.s indicates nonsignificant difference between the NoAT and Base conditions. * indicates significant difference between AT and NoAT conditions. EBR: eye blink rate, Base: baseline; NoAT: no attention; AT: attention.

The result demonstrated that EBR significantly changed following shift of visual attention. The results revealed a main effect of condition on EBR (p = 0.013). Post hoc tests revealed that AT was significantly lower than both Base (p_corr_ < 0.001) and NoAT (p_corr_ < 0.001), whereas the difference between Base and NoAT was not significant (p > 0.99) (Fig. 8). Moreover, EBR remained stable across 9 trials in the AT. The Friedman test did not reveal a significant main effect of trial order on EBR (p = 0.45). Regarding the relation between EBR and ML, the results suggested that no significant correlation was found between the MLI and ΔEBR (p = 0.39).

## Discussions

These findings support our hypothesis that increased attentional demands during the visuomotor task were reflected in enhanced SG at the cortical level, accompanied by reductions in HRV and EBR. In addition, HRV was suggested to associate with ML.

### SG as a neural correlate of attentional modulation

The findings consistently revealed a pronounced increase in SG under attentional conditions, and the magnitude of the attentional effect (NoAT → AT) was comparable across modalities. This extends previous knowledge suggesting that attention primarily modulates the prioritization of information within a given sensory channel, as demonstrated in the cocktail party effect [33] and visual dominance phenomena observed in the Colavita effect [34]. In the context of the Colavita effect, when participants perform visual target detection with auditory distractor, reaction times can be faster than during visual target detection with visual distractors. This has been interpreted as evidence that visual distractors may interfere more strongly with attentional processing than auditory distractors at the behavioral level. However, the present findings did not support such modality-specific asymmetry. This discrepancy may be explained by differences in experimental design. In previous behavioral paradigms, distractors and targets often appeared concurrently, requiring direct competition and overt selection of target responses. In contrast, the present study introduced distractors in a randomized manner while participants were already engaged in a well-established task set, with a predefined target representation. This may have reduced modality-dependent competitive interactions and led to more uniform inhibitory mechanisms across sensory systems. Taken together, that provides a holistic approach for evaluating sensory inhibition processes at the cortical level. This approach has been previously presented in studies assessing the flow state [14].

The effect of attention on SG can be explained via neural mechanisms such as limitation of attentional resources, top-down inhibitory control, and dynamic interactions between attentional networks. First, according to perceptual load theory [35], the perceptual demands of a task determine the extent to which attentional resources are available for processing task-irrelevant stimuli. Under a low perceptual load, surplus attentional capacity may be involuntarily distributed to distractors, thereby increasing susceptibility to interference. In contrast, when the perceptual load is high, attentional resources are fully engaged, effectively limiting the processing of extraneous information. Accordingly, in the AT, resources were selectively directed toward task-relevant inputs, including visual cues and proprioceptive feedback, rather than irrelevant sensory inputs. Second, top-down inhibitory control, which is mediated mainly by the dorsolateral prefrontal cortex and the posterior parietal cortex, helps focus attention by filtering out irrelevant sensory information [36]. The dorsolateral prefrontal cortex manages executive functions and goal-directed control by exerting inhibitory signals that suppress the processing of distracting stimuli [37]. Moreover, the posterior parietal cortex supports spatial attention and sensory integration, prioritizing relevant sensory inputs for further processing [38]. Third, the interaction between the dorsal attention network and the ventral attention network has been implicated. In the AT, increased dorsal network activity enhances selective attention, whereas decreased ventral network activity reduces sensitivity to irrelevant stimuli by dampening bottom-up attention [39].

More insights into individual components of cortical sensory response, our findings consistently revealed the modulation of multiple SG related to attention was observed in later components but was not in the initial component. There was no significant attentional modulation in the initial components across modalities such as in P1 (AEP), N1 (VEP), or N1 (SEP). Previous studies have shown that the initial components remain unchanged following attentional modulation in SEP [13], AEP [40], and VEP [41]. While another report revealed significant modulation of somatosensory at the first negative peak response (N1), and later components P3 when individuals engaged in an active virtual reality task [15]. This finding suggests that higher attentional demand or other psychological factors (e.g., flow state) could influence early somatosensory perception by filtering peripheral sensory information at the subcortical level [15]. Differences observed in these initial components may be related to the level of attentional engagement, which may vary across different tasks.

In contrast to the first stable component, the following components showed significant change due to shift of attention, such as in N1, P2 (AEP), P1, N2 (VEP), P2, and P3 (SEP). Previous studies have shown that attentional modulation primarily affects AEP components in N1 and P2 [42], [43], VEP components in P1 and N2 [17], and SEP components in P2 and P3 [13], [15]. These components were sensitive to shift of attention, suggesting they reflect key process to identify the basic of external stimulus features. Therefore, as attention to stimuli decreases, their amplitude significantly reduces. Given this, these changes, previously linked to attention in cognitive paradigms, were also observed in complex motor tasks in the present study, highlighting their potential for assessing dynamic changes in motor performance. However, the later components such as N2 in AEP, P2 in VEP did not significantly change across condition. This finding suggests that the shift in attention between conditions inadequately modulated processes associated with higher-order sensory functions, which corresponds to characteristics that warrant a deeper analysis of sensory signals.

Interestingly, the results revealed a significant change in the NoAT compared with the Base in P2, and P3 component of SEP, suggesting a movement-related effect on somatosensory perception, which aligns with prior research [15]. In this phenomenon, proprioceptive sensory information, including input from the joints and finger positions in space, significantly influences the processing of tactile sensory information. That may be attributed to the integration of proprioceptive and tactile sensory processing during fine motor tasks.

Overall, the findings above provide a new approach to understanding the central nervous system's response to different states of attention during motor activity compared with the approach based on analyzing cortical oscillation bands. SG offers high temporal resolution, whereas cortical oscillation bands reflect broader cognitive states, such as focus or relaxation, and offer lower temporal resolution. Thus, ERPs are ideal for studying specific cognitive responses, and cortical oscillation band analysis provides insights into an individual's overall mental state over time.

### HRV and EBR as autonomic correlates of attentional modulation

Whereas SG reflects neural mechanisms of attentional modulation at the cortical level, HRV and EBR index its autonomic and neurophysiological correlates. Within task-related HRV, the precision-demanding AT elicited systematic reductions in rMSSD, total HRV power, and spectral components, indicating diminished cardiac variability during heightened attentional engagement. These changes reflect a reorganization of cardiac autonomic modulation under increased attention demand. Decreases in rMSSD and HF power are commonly associated with reduced vagal modulation, whereas LF power represents a more complex index influenced by both autonomic and baroreflex processes [19]. Accordingly, the present findings suggest a transient adjustment in cardiac regulatory dynamics during sustained attentional focus. Importantly, this modulation occurred in parallel with enhanced SG across auditory, visual, and somatosensory modalities. The convergence of cortical (ERP-based SG) and peripheral (HRV) indices suggests that attentional engagement during visuomotor performance is accompanied by coordinated adjustments across central and autonomic systems. Although no direct neural measures were obtained, the observed pattern is consistent with theoretical frameworks proposing functional integration between higher-order attentional control networks and cardiac vagal regulation during cognitively demanding states. In particular, the neurovisceral integration model posits that prefrontal and anterior cingulate regions involved in executive attention exert regulatory influence over subcortical and brainstem structures that modulate cardiac vagal output [21]. However, given the absence of respiratory monitoring and the relatively short recording duration, the frequency-domain findings should be interpreted as reflecting short-term autonomic adjustments rather than stable shifts [44], [45].

Furthermore, across ML progression, HRV modulation varied as a function of trial order, with more pronounced reductions during early learning stages and partial attenuation as performance stabilized. This temporal dynamic suggests that autonomic adjustments track the evolving attentional demands of skill acquisition. Early stages, characterized by high cognitive control and performance monitoring, were associated with stronger reductions in cardiac variability, whereas later stages when execution becomes more efficient, showed relative recovery, consistent with previous work on mental engagement [46], or motor sequence learning [47]. Furthermore, we observed a negative correlation between the MLI and total HRV power, suggesting that individuals who exhibited smaller task-related modulation of HRV tended to demonstrate more efficient learning trajectories. These findings are consistent with previous evidence suggesting that HRV is related to cognitive–motor function. For example, higher resting HRV has been reported to correlate with motor recovery following stroke [48], and HRV biofeedback training has been shown to improve motor function through modulation of autonomic regulation [49].

Additionally, EBR also provides a reliable objective measure of visual attention. Previous studies have reported decreased EBR during cognitive activities, such as reading [25] and online learning [50]. EBR suppression may result from the prefrontal cortex mediated inhibition of blink-related motor areas, minimization of visual interruptions, and stabilization of foveal input to facilitate continuous processing. Evaluating attention through EBR presents significant potential for practical applications, particularly with the advancement of automated monitoring systems such as eye tracking and artificial intelligence-based computation. Furthermore, the integration of additional metrics, such as eye movement and pupil size [8], [50], could increase the precision of attention assessments. Although EBR is a valuable marker of visual attention, its application is limited to tasks without a visual target, such as internal attention.

However, the absence of a significant effect of trial order in the AT on EBR indicates that EBR remained stable, even though these trials reflect ongoing ML. This suggests that EBR does not track the temporal dynamics of ML, at least within the time scale examined here. In other words, while behavioral adaptation is expected to evolve across trials, the underlying blink-related processes appear to remain relatively constant. Furthermore, no significant correlation was found between MLI and ΔEBR, indicating that individual differences in ML ability were not associated with changes in EBR. This dissociation suggests that EBR may reflect a more global or tonic aspect of attentional state, rather than learning-specific processes.

### Implications

These findings highlight the potential of integrating physiological markers including SG, autonomic indices to provide objective indices of attentional engagement during motor tasks. Such measures offer reliable, quantifiable insights that complement subjective assessments of attention [51]. Continuous monitoring of these signals may help identify individual differences in attentional focus and support the design of personalized strategies to optimize skill learning and rehabilitation [52]. Additionally, combining these physiological markers with machine learning approaches could enable real-time evaluation and prediction of cognitive states during motor performance, extending beyond traditional EEG-based methods used in purely cognitive tasks [53]. By examining attention in a relatively simple visuomotor task, the study provides a framework for understanding how attentional engagement may influence everyday functional activities and structured training programs. Collectively, these results establish a foundational reference for future investigations into the role of attention in complex motor skill acquisition.

### Limitations

Several limitations should be considered. First, the sample comprised a relatively small group of young healthy adults, which may limit generalizability to older or clinical populations. Larger and more diverse samples are needed to confirm the robustness of the SG and autonomic findings. Second, HRV was derived from short recording periods (90 s) without direct respiratory monitoring. Because respiration influences vagally mediated indices, particularly HF power, frequency-domain measures likely reflect short-term task-related modulation rather than stable autonomic states. Third, although the design targeted attentional modulation, the precision-demanding condition may also have increased general task difficulty. Thus, the physiological changes cannot be attributed solely to attention. Fourth, correlations with MLI were largely exploratory; except for total HRV, associations did not survive correction for multiple comparisons. Causal relationships cannot be inferred. Finally, while ERP-based SG offers high temporal resolution, the limited electrode montage restricted spatial interpretation of cortical sources. Future studies using high-density EEG or multimodal imaging may better delineate the underlying networks.

## Conclusion

The present study highlights SG as a neural correlate of attentional modulation during a visuomotor typing task. Attentional demands were observed in specific ERP components across sensory modalities at later components (N1, P2 in AEP; P1, N2 in VEP; P2, P3 in SEP), indicating systematic modulation of sensory processing. In addition to these neural effects, autonomic and neurophysiological indices, including HRV parameters (rMSSD, LF power, HF power, total HRV power) and EBR showed task-related changes. Notably, HRV exhibited dynamic fluctuations across the ML process and was associated with ML improvement. Together, these findings extend our understanding of how attentional processes modulate multiple physiological indices during complex motor activity.

## CRediT authorship contribution statement

**Cuong Nguyen Van:** Writing – review & editing, Writing – original draft, Visualization, Validation, Software, Methodology, Investigation, Formal analysis, Data curation, Conceptualization. **Duc Le Trung:** Visualization, Methodology, Conceptualization. **Takeru Okamoto:** Writing – review & editing, Methodology, Investigation. **Masataka Tatsumi:** Writing – review & editing, Methodology. **Thu Nguyen Dang:** Writing – review & editing, Methodology, Conceptualization. **Yuya Hyodo:** Writing – review & editing, Methodology, Investigation. **Satoshi Kase:** Writing – review & editing, Methodology, Investigation. **Tomoyuki Kurose:** Writing – review & editing, Supervision, Methodology, Conceptualization. **Takeshi Imura:** Writing – review & editing, Supervision, Methodology, Investigation, Conceptualization. **Hisao Nishijo:** Writing – review & editing, Supervision, Conceptualization. **URAKAWA SUSUMU:** Writing – review & editing, Writing – original draft, Visualization, Validation, Supervision, Software, Resources, Project administration, Methodology, Funding acquisition, Conceptualization.

## Funding

This research was funded by the Japanese Ministry of Education, Culture, Sports, Science, and Technology through a Grant-in-Aid for Scientific Research (23K10503 to SU) and supported by research grants (0G4170) from 10.13039/501100003790Hiroshima University.

## Declaration of Competing Interest

The authors declare that they have no known competing financial interests or personal relationships that could have appeared to influence the work reported in this paper.
