# Supplementary material for: Attentional modulation of sensory gating during a visuomotor task
Source: J Physiol Sci. 2026 May 15;76(2):100080. doi: 10.1016/j.jphyss.2026.100080 (PMC13199777; doi:10.1016/j.jphyss.2026.100080)
Supplement: Supplementary file 1 — Supplementary material [file mmc1.docx]

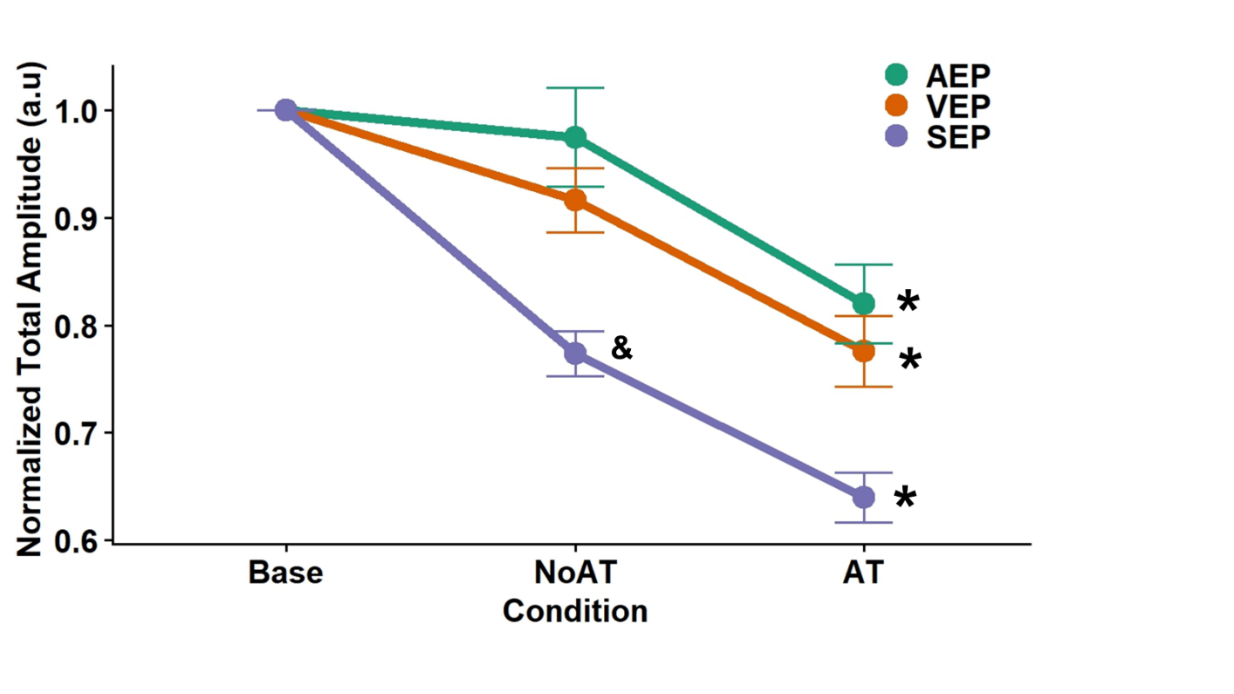


**Figure S1. Effects of condition x modality interaction on SG across modalities.** Data were normalized relative to the Base to account for baseline differences between modalities. A linear mixed-effects model was used to examine the main effect of the condition × modality interaction. Post hoc paired t-tests with Bonferroni correction indicated significant differences between conditions. “*” denotes a significant difference between AT and NoAT, and “&” denotes a significant difference between NoAT and Base (p*<* 0.05). AEP: auditory evoked potential; VEP: visual evoked potential; SEP: somatosensory evoked potential; SG: sensory gating; Base: baseline condition; NoAT: no attention condition; AT: attention condition
