# Supplementary material for: Attentional modulation of sensory gating during a visuomotor task
Source: J Physiol Sci. 2026 May 15;76(2):100080. doi: 10.1016/j.jphyss.2026.100080 (PMC13199777; doi:10.1016/j.jphyss.2026.100080)
Supplement: Supplementary file 2 — Supplementary material [file mmc2.docx]

**
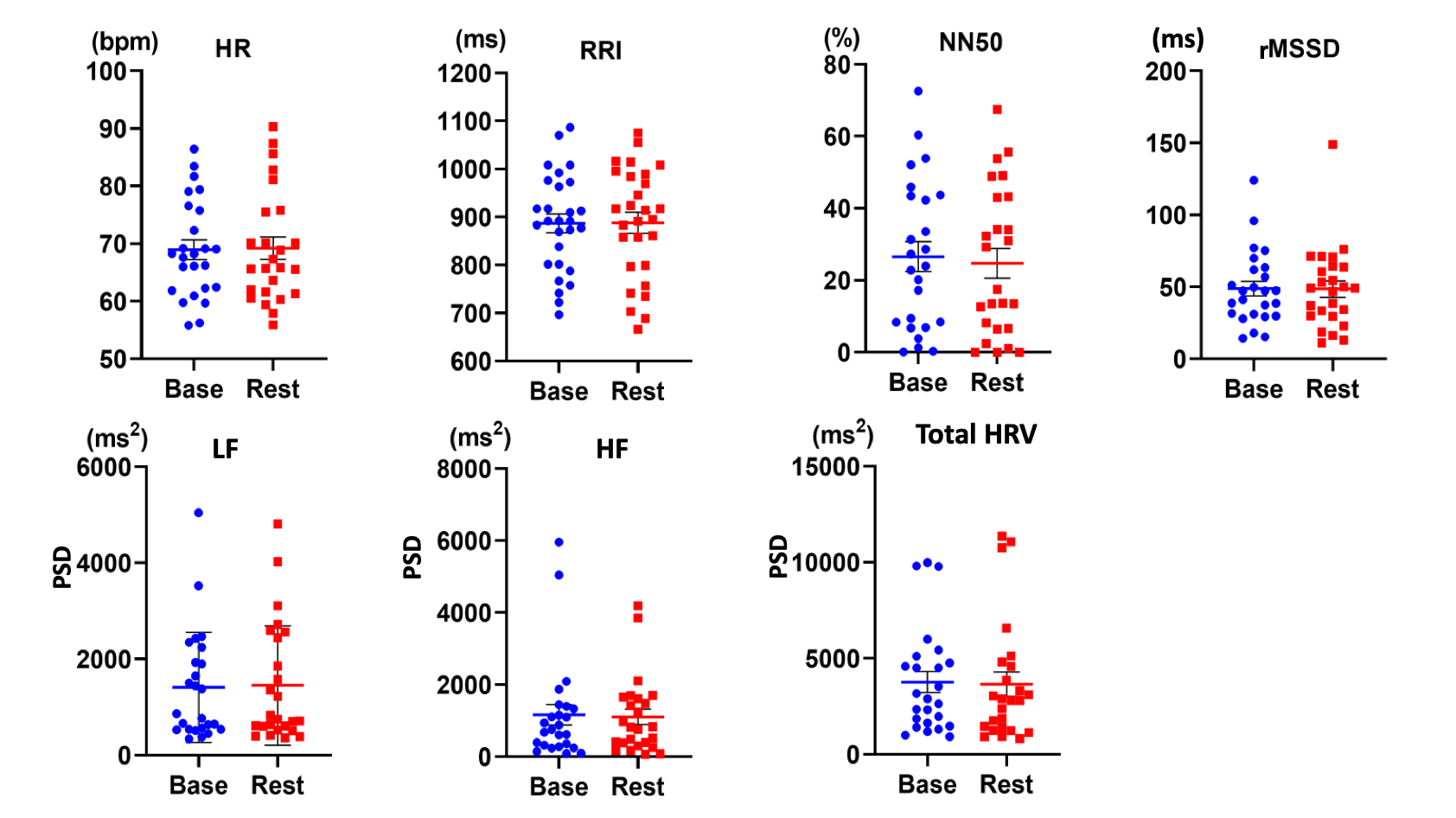
**

**Figure S2.** Comparison of HRV indices between the resting state (Rest) and the Base. The Wilcoxon signed-rank test was used to test the difference between two conditions. No significant differences were observed across all measures. The data are presented as means ± standard errors of the means. HF: high-frequency; HRV: heart rate; HRV: heart rate variability; LF: low-frequency; NN50: number of successive R–R intervals differing by more than 50 ms; PSD: power spectral density; RRI: **R–R interval**; rMSSD: root mean square of successive differences.
